# Sexual orientation disparities in the co-occurrence of substance use and psychological distress: a national population-based study (2008–2015)

**DOI:** 10.1007/s00127-018-1491-4

**Published:** 2018-02-15

**Authors:** Richard Bränström, John E. Pachankis

**Affiliations:** 1 Department of Social and Behavioral Sciences, Yale School of Public Health, New Haven, CT USA; 2 Department of Clinical Neuroscience, Karolinska Institutet, Nobels väg 9, 171 77 Stockholm, Sweden

**Keywords:** Sexual minorities, Substance use, Psychological distress, Syndemic, Minority stress

## Abstract

**Purpose:**

Although strong evidence documents the elevated prevalence of both substance use and mental health problems among sexual minorities (i.e., gay, lesbian, and bisexuals), relatively less research has examined whether risk of the co-occurrence of these factors is elevated among sexual minorities compared to heterosexuals. The object of this study was to (1) explore sexual orientation-based differences in substance use, psychological distress, and their co-occurrence in a representative sample in Sweden, and (2) examine if greater exposure to stressors, such as discrimination, victimization/threats, and social isolation, could explain these potential disparities and their co-occurrence.

**Methods:**

Data come from the cross-sectional Swedish National Public Health Survey, which collected random samples of individuals (16–84 years of age) annually from 2008 to 2015, with an overall response rate of 49.7% (*n* = 79,568 individuals; 1673 self-identified as lesbian, gay, or bisexual). Population-level sexual orientation differences in substance use (i.e., alcohol, tobacco, and cannabis) and psychological distress were examined.

**Results:**

Our findings showed significantly elevated prevalence of high-risk alcohol use, cannabis use, and daily tobacco smoking, among sexual minorities compared to heterosexuals. These substantial disparities in substance use more often co-occurred with psychological distress among sexual minorities than among heterosexuals. The elevated risk of co-occurring psychological distress and substance use was most notable among gay men relative to heterosexual men (adjusted odds ratio [AOR] = 2.65, CI 1.98, 3.55), and bisexual women relative to heterosexual women (AOR = 3.01, CI 2.43, 3.72). Multiple mediation analyses showed that experiences of discrimination, victimization, and social isolation partially explained the sexual orientation disparity in these co-occurring problems.

**Conclusions:**

This study adds to a growing body of research showing that sexual minorities experience multiple threats to optimal health and points toward future interventions that address the shared sources of these overlapping health threats in stigma-related stress.

## Introduction

Recent studies have shown significant sexual orientation differences in the prevalence of alcohol abuse, recreational drug use, and tobacco smoking, with sexual minorities (i.e., those self-identifying as lesbian, gay, or bisexual [LGB] or those reporting same-sex sexual experiences) reporting greater substance use than heterosexuals [1–8]. Large differences also exist in mental health problems such as depression, anxiety disorders, and suicide attempts between sexual minorities and heterosexuals [9–15]. These disparities in substance use and mental health problems emerge early in development, persists across the life course, and expose sexual minorities to a greater risk of potentially avoidable diseases than heterosexuals [16–20].

Although strong evidence documents the elevated prevalence of both substance use and mental health problems among sexual minorities, relatively less research has examined whether risk of the co-occurrence of these factors is elevated among sexual minorities compared to heterosexuals [21]. However, studies suggest that poor mental health and substance use frequently co-occur among sexual minority individuals [21–23], potentially due to their shared source in sexual minorities’ exposure to stigma [24].

Stigma, including exposure to unequal treatment and other forms of discrimination, has been argued to represent a fundamental cause of adverse health conditions [25]. Stigma compromises health, because it compromises access to the knowledge, prestige, power, and supportive social connections necessary to prevent disease [20]. Notably, stigma as a fundamental cause of poor health affects not just isolated disease conditions, but many. A complementary theory of poor health among stigmatized populations—syndemic theory—suggests that, against stigmatizing social backdrops, certain disease epidemics co-occur and perpetuate each other [26]. In fact, studies have shown that sexual minorities experience higher rates of depression, substance use disorder, suicidality, and exposure to violence, and that these adverse outcomes are associated with stigma [27–32].

In the case of sexual minority men, accumulating evidence suggests that structural stigma in the form of discriminatory laws and policies toward sexual minorities, interpersonal stigma in the form of victimization and discrimination, and intrapersonal stigma in the form of internalized homophobia and anxious expectations of rejection are all associated with substance use and mental health problems [6, 18, 33–44]. However, less clear is whether risk of the co-occurrence of substance use and mental health problems is elevated among sexual minorities compared to heterosexuals and whether sexual minorities’ disproportionate exposure to stigma and discrimination explain their increased risk of these co-occurring health risks.

The present study takes advantage of the data available within the representative population-based Swedish National Public Health Survey conducted between 2008 and 2015. Pooling eight consecutive years of survey data provides a large enough sample size to address existing gaps in knowledge regarding sexual orientation disparities in substance use and co-occurring mental health problems. Specifically, the large sample size and representative data structure permitted us to pursue the following research questions: (a) are substance use (i.e., high-risk alcohol use, use of cannabis, and daily smoking) and psychological distress more prevalent among sexual minority individuals than among heterosexuals? (b) Is the co-occurrence of substance use and psychological distress more common among sexual minorities than among heterosexuals; and (c) can greater exposure to stressors, such as discrimination, victimization/threats, and social isolation, explain or partially explain the elevated prevalence of substance use, psychological distress, and their co-occurrence among sexual minorities? (d) Are sexual orientation disparities in co-occurring substance use and psychological distress greater for bisexuals compared to gays/lesbians, for sexual minority men compared to women, and for adolescent/young adult sexual minorities compared to adult sexual minorities?

## Methods

### Participants

Each year between 2008 and 2015, the Public Health Agency of Sweden collected nationwide population-based health surveys in independent unrestricted random samples of the general population in Sweden (20,000 individuals each year), ages 16–84. A total of 79,568 individuals responded across the eight surveys. Identical modes of data collection, questions, and survey administration were used in all years and participants were offered the option to respond to the questions via either paper-and-pencil mailed questionnaires or self-administered web surveys. Data from each annual survey were pooled into one data set. The response rate varied between 48.1 and 60.8% each year with an overall response rate of 49.7%. The response rate was higher among women and older age groups. In addition to a question regarding sexual orientation, the survey assessed socio-demographic background, health status, and health determinants, and was supplemented with data from administrative national registries regarding income, ethnicity, and urbanicity. The study was approved by the Regional Ethics Committee in Stockholm (No. 2013/2200-31/2).

### Measures

*Sexual orientation* Individuals’ sexual orientation was classified based on self-identification using the following item: “What is your sexual orientation?” with the response categories: “heterosexual,” “bisexual,” “homosexual,” and “not sure.” The rate of non-response for this question has continually decreased, from 6.9% in 2008 to 3.2% in 2015. In the total sample, 588 (0.7%) individuals self-identified as gay men/lesbian and 1085 (1.4%) self-identified as bisexual. The proportion of respondents reporting an LGB identity remained stable over the 10-year period (the range for gay men/lesbian was 0.7–0.8% and for bisexuals 1.2–1.8%). We excluded 1382 (1.7%) individuals who responded that they were uncertain of their sexual orientation, as previous studies have shown that this group often consists of a heterogeneous mix of respondents in terms of sexual identity [45]. While some people do not know their sexual orientation because they are undecided, studies have indicated that the majority of people who choose such responses in population surveys are doing so because they did not understand the question [46]. Those who responded that they were “not sure” of their sexual orientation did not differ significantly regarding gender and age, but were more often born outside of Sweden, had lower income and education, were less often married or partnered, and were more likely to report poor mental health as compared to those reporting being heterosexual (all associations were significant at *P* < 0.001).

*Socio-demographic variables* Socio-demographic factors, including age, annual individual income, ethnicity (i.e., nation of birth categorized into geographic regions), relationship status, and urbanicity (i.e., living in larger city, smaller city, or rural community), were collected from national registries and linked to the questionnaire data, which also included self-reported relationship status (i.e., living with partner versus single). These covariates were chosen, because they were significantly associated with sexual orientation and with psychological distress in bivariate models and could therefore serve as potential confounders.

*Substance use* In the current study, three forms of substance use were analyzed: high-risk alcohol consumption, cannabis use, and tobacco use. All of these were coded as dichotomous variables. Two different measures were used to categorize respondents into high-risk versus non-high-risk consumers of alcohol. The first concerned average frequency of heavy drinking during the past 12 months, based on one question regarding drinking at least one bottle of wine or equivalent during one occasion. The second measure concerned total weekly amount of alcohol consumed on average during the past 12 months, measured as number of “drinks” (defined as 33 centiliters [cl] of beer, 10–15 cl of wine, 4 cl of hard liquor, or equivalent). Male respondents were categorized as high-risk consumers of alcohol if they either reported at least monthly heavy alcohol consumption or reported an average weekly consumption of more than 14 drinks, in accordance with the threshold for hazardous weekly alcohol consumption proposed by the Swedish National Institute of Public Health [47]. Women were similarly categorized as high-risk consumers of alcohol if they either reported at least monthly heavy alcohol consumption or reported an average weekly consumption of more than nine drinks. Cannabis use was assessed with one question regarding frequency of cannabis use during the past 12 months, which was categorized based on any past-12-month use (use/no use). One question regarding daily tobacco smoking was used to categorize the respondents into current daily smokers versus non-smokers.

*Psychological distress* The 12-item General Health Questionnaire (GHQ12) was used to assess recent symptoms of psychological distress. The GHQ12 is a frequently used measure of current mental health and focuses on two major types of symptoms: anhedonia (e.g., “Over the past few weeks, have you been able to enjoy your normal day-to-day activities?” with response alternatives: ‘more so than usual’; ‘same as usual’; ‘less so than usual’; and ‘much less than usual’) and depressed mood (e.g., “Over the past few weeks, have you been feeling unhappy and depressed?” with response alternatives: ‘not at all’; ‘no more than usual’; ‘rather more than usual’; and ‘much more than usual’). It has shown adequate validity in both clinical and general populations and has demonstrated satisfactory sensitivity and specificity for predicting current diagnosis of major depression [48, 49]. Consistent with prior research [49], we created a dichotomous variable (GHQ12 ≤ 3: ‘no current psychological distress’; GHQ12 ≥ 4: ‘current psychological distress’).

*Stress exposure* Three questions were used to assess exposure to discrimination, victimization/threats, and social isolation. Perceived discrimination was assessed with one question: “During the past three months, have you been treated in a way that made you feel discriminated against [yes/no]?” Two questions assessed experiences of victimization and threat of assaults: “During the past 12 months, have you ever been physically assaulted [yes/no]?” and “During the past 12 months, have you ever been exposed to threats or threats of violence, severe enough to make you scared [yes/no]?” Respondents were categorized as having versus not having been exposed to discrimination or victimization. Two questions were used to assess social isolation—one question regarding emotional social support: “Do you have anyone you can share your innermost feelings with and confide in [yes/no]?” and one question regarding instrumental social support: “Can you get help from any person or persons if you have practical problems or are ill? e.g. get advice, borrow things, help with shopping, repairs etc. [yes/no].” Respondents were categorized as socially isolated if they lacked both emotional and instrumental social support or not socially isolated if they reported at least one type of support.

### Statistical analysis

After examining descriptive statistics, sexual orientation differences in substance use and psychological distress were analyzed using logistic regression. Post-stratification weights were used to adjust for selection probabilities and non-response to generate nationally representative estimates of prevalence and associations. The analyses were adjusted for age, income, education, urbanicity, relationship status, and country of birth. Dummy variables were created so as to indicate the presence of a combination of any substance use (high-risk alcohol consumption, use of cannabis, and daily smoking) and elevated psychological distress. We then used these dummy variables to explore sexual orientation differences in the co-occurrence of substance use and psychological distress using logistic regression. Only respondents with complete data on each outcome variable were included in analyses, but missing values on outcome variables were infrequent and varied between 0.6% (tobacco use) and 2.5% (psychological distress).

We then examined whether stress exposure (i.e., discrimination, victimization/threats, social isolation) explains or partially explains elevated prevalence of substance use, psychological distress, and their co-occurrence among sexual minorities. We conducted three separate multiple mediation analyses, one for each outcome variables. For all three multiple mediation analyses, all three proposed mediating variables were included (i.e., discrimination, victimization, and social isolation). To statistically test the mediating effects, we calculated the indirect effects of exposure to stressors (i.e., discrimination, victimization/threats, and social isolation) as mediators of the link between sexual orientation and our outcomes. A significant indirect effect (*P* < 0.05) was interpreted as evidence of mediation.

In addition to the main analyses, effect modification by gender and age was examined in secondary subgroup analyses. Stratified analyses were performed if the interaction between sexual orientation and these variables was significant.

Data analyses were conducted using both SPSS (version 24) and Mplus (version 8).

## Results

### Descriptive statistics

Table 1 presents socio-demographic characteristics and exposure to stressors in the total sample and stratified by sexual orientation. Gay men/lesbians were more likely to be male, younger, university educated, born outside of Sweden, living in a larger city, and living without a partner, and have lower income, as compared to heterosexuals. Bisexuals were more likely to be female, younger, born outside of Sweden, living in a larger city, living without a partner, and having lower income, compared to heterosexuals. All associations between socio-demographic factors and sexual orientation were significant at *P* < 0.001 using Chi square tests for categorical variables and *t* test for continuous variables. Concerning exposure to stress, both sexual minority groups (i.e., gay men/lesbians and bisexuals) reported greater exposure to discrimination and victimization/threats during the past 12 months than heterosexuals; and they were more likely to report being socially isolated (all *P* < 0.001).

**Table 1:** Socio-demographics, psychological distress, substance use, and exposure to stressors by sexual orientation in the Swedish National Public Health Survey, between 2008 and 2015

	Total, *n* = 78,052	Heterosexual, *n* = 76,379 (97.8%)	Gay men/lesbian, *n* = 588 (0.8%)	Bisexual, *n* = 1085 (1.4%)
	%^a^	%^a^	%^a^	%^a^
Gender				
Men	50.3	50.4	66.2	36.9
Women	49.7	49.6	33.8	63.1
Age				
16–24 years	14.1	13.8	18.1	31.9
25–34 years	14.7	14.5	24.7	25.7
35–46 years	20.9	20.8	30.8	20.5
47–84 years	50.2	50.9	26.4	21.9
Education				
Less than university	65.6	65.8	53.6	64.6
University degree	34.4	34.2	46.4	35.4
Income				
1 lowest quartile	24.0	23.6	30.4	45.3
2 second lowest	24.3	24.3	22.5	24.1
3 second highest	25.7	25.8	25.0	18.1
4 highest quartile	26.0	26.3	22.1	12.5
Relationship status				
Married/partnered	64.6	65.0	50.1	45.6
Single	35.4	35.0	49.9	54.4
Country of birth				
Sweden	85.3	85.5	72.9	79.7
Other country	14.7	14.5	27.1	20.3
Urbanicity				
Larger city	32.0	31.7	54.2	39.1
Smaller city	32.3	32.4	26.5	30.3
Rural community	35.7	36.0	19.3	30.6
Psychological distress				
GHQ12 ≥ 4	11.9	11.7	18.2	28.1
Substance use				
High-risk alcohol consumption	16.5	16.4	25.2	20.3
Past-12-month use of cannabis	1.5	1.4	4.3	7.7
Daily tobacco smoking	10.7	10.6	15.2	17.1
Exposure to stressors				
Discrimination	19.4	19.2	33.3	39.7
Victimization/threats	5.8	5.6	9.0	13.9
Social isolation	4.6	4.5	10.5	9.1

^a^Weighted percentages

### Sexual orientation disparities in substance use, psychological distress, and co-occurring substance use and psychological distress

Table 2 presents sexual orientation disparities in substance use and psychological distress. Gay men/lesbians had elevated risk of high-risk alcohol consumption, cannabis use, daily tobacco smoking, and psychological distress compared to heterosexuals. Bisexuals had elevated risk of cannabis use, daily smoking, and psychological distress compared to heterosexuals. Furthermore, the last column of Table 2 presents sexual orientation disparities in co-occurring substance use and psychological distress. Both gay men/lesbians and bisexuals had elevated risk of co-occurring psychological distress and substance use compared to heterosexuals.

**Table 2:** Adjusted odds ratios for sexual orientation disparities in substance use, psychological distress, and their co-occurrence

	High-risk alcohol consumption	Past-12-month use of cannabis	Daily tobacco smoking	Psychological distress (GHQ12 ≥ 4)	Co-occurring psychological distress and substance use
	AOR (95% CI)	AOR (95% CI)	AOR (95% CI)	AOR (95% CI)	AOR (95% CI)
Heterosexual (reference)	1	1	1	1	1
Gay men/lesbian	1.33** (1.11, 1.58)	1.91*** (1.37, 2.66)	1.72*** (1.39, 2.12)	1.52*** (1.25, 1.85)	2.07*** (1.60, 2.69)
Male	–	–	2.30*** (1.80, 2.93)	–	2.65*** (1.98, 3.55)
Female	–	–	0.76 (0.48, 1.20)	–	0.96 (0.52, 1.76)
16–34 years	–	–	–	2.03*** (1.55, 2.66)	–
35–84 years	–	–	–	1.12 (0.83, 1.50)	–
Bisexual	1.13 (0.98, 1.30)	2.73*** (2.19, 3.40)	1.86*** (1.60, 2.16)	2.35*** (2.06, 2.67)	2.63*** (2.20, 3.14)
Male	0.95 (0.76, 1.18)	1.75** (1.22, 2.50)	-	1.92*** (1.52, 2.44)	1.88*** (1.35, 2.60)
Female	1.27* (1.06, 1.54)	3.42*** (2.58, 4.53)	-	2.48*** (2.12, 2.90)	3.01*** (2.43, 3.72)
16–34 years	–	–	2.29*** (1.88, 2.79)	–	3.07*** (2.49, 3.78)
35–84 years	–	–	1.32* (1.04, 1.68)	–	1.96*** (1.40, 2.76)

All models are adjusted for age, gender, education, income, relationship status, country of birth, and living in urban or rural communities. All models take into account sample weights. Analyses were stratified by gender or age if test of interactions were significant at *P* < 0.05

*AOR* adjusted odds ratios, *CI* confidence interval

* Significant at *p* < 0.05

** Significant at *p* < 0.01

*** Significant at *p* < 0.001

### Stress exposure as a mediator of sexual orientation disparities in substance use, psychological distress, and their co-occurrence

**Fig. 1:**
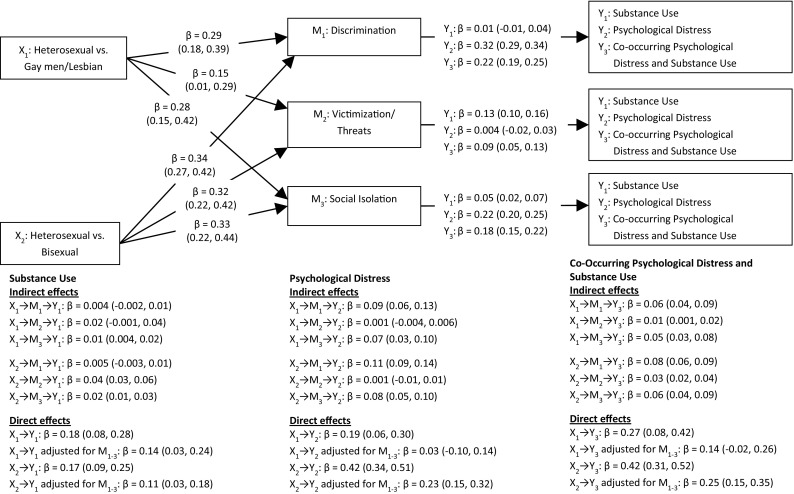
Indirect effect of sexual orientation on substance use, psychological distress, and their co-occurrence through individual/interpersonal-level mediators

To explore the indirect effect of stress exposure on substance use, psychological distress, and their co-occurrence, a set of serial multiple mediation analyses was conducted (Fig. 1). The analyses showed significant indirect mediating effects of social isolation in the association between sexual orientation and substance use among gay men/lesbians, and of both social isolation and victimization/threats among bisexuals. The analyses also showed significant indirect mediating effects of both discrimination and social isolation in the association between sexual orientation and psychological distress among gay men/lesbians and bisexuals. Furthermore, the associations between sexual orientation and the co-occurrence of substance use and psychological distress were significantly mediated through all three mediators (i.e., discrimination, victimization, and social isolation). The effects were all in the same direction, indicating that the elevated risk of substance use, psychological distress, and their co-occurrence among sexual minorities compared to heterosexuals, could partially be explained by sexual minorities’ elevated exposure to discrimination, victimization/threat, and social isolation.

### Effect modification by gender and age

To determine if sexual orientation disparities in substance use, psychological distress, and their co-occurrence differed by gender and age, analyses of interactions between sexual orientation and gender, as well as sexual orientation and age, were performed. For significant effects, stratified analyses are presented in Table 2. The prevalence of substance use, psychological distress, and their co-occurrence is presented in Table 3 stratified by sexual orientation, gender, and age. Gender and age were found to be a significant effect modifier of several outcomes. In stratified analyses, the sexual orientation disparity in high-risk alcohol consumption was only significant among bisexual, compared to heterosexual, women, but not for bisexual, compared to heterosexual, men. Further, the sexual orientation disparity in cannabis use was greater among bisexual, compared to heterosexual, women than among bisexual, compared to heterosexual, men. The sexual orientation disparity in daily tobacco smoking was only significant among gay, compared to heterosexual, men but not among lesbian, compared to heterosexual, women. The sexual orientation disparity in psychological distress was greater among bisexual, compared to heterosexual, women than among bisexual, compared to heterosexual, men. Gender-stratified analyses of co-occurring psychological distress and substance use showed that the elevated risk of this co-occurrence was only present among gay men compared to heterosexual men, and not among lesbian women compared to heterosexual women. Further, the elevated prevalence of co-occurring substance use and psychological distress was larger among bisexual women compared to heterosexual women than among bisexual men compared to heterosexual men.

**Table 3:** Prevalence of substance use, psychological distress, and their co-occurrence by sexual orientation, gender, and age

	Men			Women		
	Heterosexua *n* = 34,921	Gay *n* = 355	Bisexua *n* = 369	Heterosexual *n* = 41,458	Lesbian *n* = 233	Bisexual *n* = 7116
	%^a^ (95% CI)	%^a^ (95% CI)	%^a^ (95% CI)	%^a^ (95% CI)	%^a^ (95% CI)	%^a^ (95% CI)
High-risk alcohol consumption						
16–34 years	32.2 (31.4, 33.1)	37.7 (30.8, 44.6)	29.0 (22.8, 35.2)	18.4 (17.6, 19.1)	15.2 (8.4, 22.0)	23.3 (19.6, 27.0)
35–84 years	19.5 (19.0, 20.0)	25.9 (20.7, 31.2)	22.4 (17.1, 27.7)	10.0 (9.7, 10.4)	22.2 (14.8, 29.6)	10.7 (7.0, 14.4)
Past-12-month use of cannabis						
16–34 years	7.8 (7.3, 8.3)	12.0 (7.4, 16.6)	14.9 (10.0, 19.8)	3.9 (3.5, 4.3)	7.1 (2.2, 11.9)	12.7 (9.8, 15.6)
35–84 years	0.9 (0.8, 1.1)	4.0 (1.6, 6.4)	2.3 (0.4, 4.2)	0.1 (0.1, 0.2)	–	2.1 (0.4, 3.8)
Daily tobacco smoking						
16–34 years	7.0 (6.6, 7.5)	14.3 (9.3, 19.2)	18.7 (13.3, 24.0)	10.5 (9.9, 11.1)	10.1 (4.4, 15.8)	20.3 (16.8, 23.8)
35–84 years	11.1 (10.7, 11.4)	23.7 (18.6, 28.8)	19.8 (14.8, 24.7)	13.0 (12.6, 13.4)	8.3 (3.5, 13.1)	14.6 (10.4, 18.9)
Psychological distress (GHQ12 ≥ 4)						
16–34 years	11.7 (11.1, 12.3)	25.3 (19.1, 31.6)	24.1 (18.1, 30.1)	19.1 (18.3, 19.8)	26.5 (18.0, 35.0)	38.5 (34.2, 42.8)
35–84 years	9.3 (9.0, 9.6)	14.4 (10.2, 18.6)	16.1 (11.5, 20.8)	12.5 (12.1, 12.8)	12.0 (6.2, 17.8)	26.4 (21.1, 31.8)
Co-occurring psychological distress and substance use						
16–34 years	5.3 (4.9, 5.8)	16.6 (11.3, 22.0)	12.7 (8.1, 17.4)	6.2 (5.8, 6.7)	7.3 (2.3, 12.3)	19.6 (16.0, 23.1)
35–84 years	3.4 (3.2, 3.6)	8.8 (5.4, 12.2)	6.8 (3.6, 10.0)	3.3 (3.1, 3.5)	3.6 (0.3, 6.9)	8.9 (5.4, 12.3)

*CI* confidence interval

^a^Weighted percentages and confidence intervals

The sexual orientation disparity in psychological distress was greater among younger (16–34 years), compared to older (35–84 years), gay men/lesbians. The disparity in co-occurring substance use and psychological distress was also stronger among younger, compared to older, bisexuals.

## Discussion

To our knowledge, this study represents the first population-based study to examine sexual orientation disparities in the co-occurrence of substance use and psychological distress. Our data, which came from national population surveys in Sweden repeated annually between 2008 and 2015, showed significantly elevated prevalence of high-risk alcohol use, cannabis use, and daily tobacco smoking among sexual minorities than among heterosexuals. Further, these substantial disparities in substance use more often co-occurred with psychological distress among sexual minorities than among heterosexuals. The elevated risk of co-occurring psychological distress with substance use was most notable among gay men relative to heterosexual men, and bisexual women as compared to heterosexual women. No such elevated risk existed among lesbian, compared to heterosexual, women.

In addition to showing evidence of elevated co-occurring prevalence of substance use and psychological distress among sexual minorities, our findings indicate that experiences of discrimination, victimization, and social isolation partially explain the sexual orientation disparity in these co-occurring problems. Experiences of discrimination, victimization, and social isolation were elevated among both gay men/lesbians and bisexuals, and partially explained the elevated prevalence of substance use, psychological distress, as well as the co-occurrence of substance use and psychological distress among sexual minorities compared to heterosexuals.

These results support two primary tenets of syndemic theory as applied to sexual minority health [26, 32], namely that psychosocial health conditions co-occur among this marginalized group and that this co-occurrence is at least partially explained by sexual minorities’ disproportionate exposure to social stressors. This study also offers some of the first evidence of syndemic theory applied to sexual minority women and suggests that co-occurring substance use and psychological distress are particularly likely to affect bisexual women. In fact, bisexual women evinced more than a three-time greater risk of co-occurring psychological distress and substance use than heterosexual women.

Other subgroup analyses partially confirm the increased disparity in substance use experienced by bisexual men and women compared to gay men/lesbians and heterosexuals reported elsewhere [7, 8, 50, 51], but expand these findings by showing an increased risk of co-occurring substance use and psychological distress. In contrast with previous findings, we did not find a gender difference in the sexual orientation disparity in high-risk alcohol consumption among gay men and lesbians [7, 8]. However, consistent with previous research, we found that the sexual orientation disparity in psychological distress is stronger among younger individuals; the present study finds that this age pattern extends to the co-occurrence of substance use and psychological distress, but only for bisexuals [52].

Results should be considered in light of several limitations. First, given that data were collected cross-sectionally at each assessment point, we are unable to establish the causal direction of effects. It is possible that their higher engagement in substance use puts sexual minority individuals at greater risk of exposure for discrimination and victimization, which subsequently confers risk for psychological distress. Only a prospective cohort design would allow future researchers to determine the temporal influence of minority stress exposure on substance use and its co-occurrence with psychological distress. Second, limited psychosocial measures in the datasets prevented the examination of additional potential mediators of the disparities found here. For instance, perhaps substance use coping motives or norms are particularly likely to explain the co-occurrence of substance use and psychological distress among certain sexual minority subgroups [53, 54]. Third, pooling data across several years also has limitations in that a small subset of individuals might have been included in more than one round of data collection However, we consider it unlikely that this limitation influenced our overall conclusions given the extremely low probability of being repeatedly sampled into one of the annual Swedish National Public Health Surveys. Finally, as with all population-based surveys, the current study also contained a substantial proportion of non-responders. It is possible that those who decided not to participate in the study differed systematically from those who participated. However, the primary aims of the current study were to understand sexual orientation disparities in psychological distress, substance use, and their co-occurrence, as well as psychosocial mediators of these disparities, and we consider it unlikely that these aims are substantially influenced by a biased selection of participants by sexual orientation. In support of this assumption, a previous independent population-based public health survey in southern Sweden showed a very similar proportion of sexual minorities as was found in the current study [55, 56].

Despite these limitations, the study also has several strengths, including its use of a large national population-based representative sample that included a measure of sexual orientation alongside measures of stress exposures and behavioral health outcomes. Nationally representative datasets that contain this combined information are rare. Consequently, many studies of sexual orientation health disparities have relied on nonrandom samples, which limits generalizability of findings [57], or have used representative samples to identify sexual orientation disparities in behavioral health outcomes but without measures of potential mediators of those disparities. The present data not only allowed us to establish a robust estimate of the prevalence of co-occurring psychosocial health outcomes in the Swedish population, but also enables a test of potential stress-related mechanisms of these co-occurring disparities as hypothesized by minority stress theory [57]. The results highlight the utility of concurrently addressing co-occurring psychosocial syndemic factors and their stigma-related stress determinants in primary and secondary prevention interventions [58].

Whereas prior research demonstrates that sexual minorities experience significant elevations in adverse psychosocial health outcomes, the present results extend those findings by showing that sexual minorities also experience elevations in the co-occurrence of these outcomes as a function of their exposure to stigma-related stressors. Future research can build on these findings by testing the causal sequence from stress exposure to these co-occurring outcomes and the potential causal influence of these outcomes on each other. Further, given that the social climate surrounding sexual minorities varies widely across world countries [59–61], future studies should also explore the extent to which the current findings generalize across countries. Such studies, for instance, might take advantage of the wide variation in social climates surrounding sexual minorities to predict sexual orientation disparities in psychosocial health outcomes and mediators from this variation. In the meantime, this study adds to a growing body of research showing that sexual minorities experience multiple threats to optimal health and points toward future interventions that address the shared sources of these overlapping health threats in social disadvantage.
